# Initial microbiome and tree root status structured the soil microbial community discrepancy of the subtropical pine-oak forest in a large urban forest park

**DOI:** 10.3389/fmicb.2024.1391863

**Published:** 2024-05-31

**Authors:** Kai Tian, Shaoming Chen, Rumeng Ye, Yanghe Xie, Lunguang Yao, Hong Lin

**Affiliations:** ^1^Henan Field Observation and Research Station of Headwork Wetland Ecosystem of the Central Route of South-to-North Water Diversion Project, School of Life Sciences and Agricultural Engineering, Nanyang Normal University, Nanyang, China; ^2^School of Life Sciences, Anhui Agricultural University, Hefei, China; ^3^School of Food Science, Institute of Applied Ecology, Nanjing Xiaozhuang University, Nanjing, China

**Keywords:** forest succession, plant–soil interaction, community assembly, microbial association, network structure, indicator species

## Abstract

Plant–microbe–soil interactions control over the forest biogeochemical cycling. Adaptive plant–soil interactions can shape specific microbial taxa in determining the ecosystem functioning. Different trees produce heterogeneous soil properties and can alter the composition of soil microbial community, which is relevant to the forest internal succession containing contrasting stand types such as the pine-oak forests. Considering representative microbial community characteristics are recorded in the original soil where they had adapted and resided, we constructed a soil transplant incubation experiment in a series of *in situ* root-ingrowth cores in a subtropical pine-oak forest, to simulate the vegetational pine-oak replacement under environmental succession. The responsive bacterial and fungal community discrepancies were studied to determine whether and how they would be changed. The pine and oak forest stands had greater heterogeneity in fungi composition than bacteria. Original soil and specific tree root status were the main factors that determined microbial community structure. Internal association network characters and intergroup variations of fungi among soil samples were more affected by original soil, while bacteria were more affected by receiving forest. Specifically, dominant tree roots had strong influence in accelerating the fungi community succession to adapt with the surrounding forest. We concluded that soil microbial responses to forest stand alternation differed between microbiome groups, with fungi from their original forest possessing higher resistance to encounter a new vegetation stand, while the bacteria community have faster resilience. The data would advance our insight into local soil microbial community dynamics during ecosystem succession and be helpful to enlighten forest management.

## 1 Introduction

Climate and environmental changes are causing increasing natural and anthropogenic disturbances on forest dynamics, which have major ecological and societal impacts (Anderson-Teixeira et al., 2013; Reyna et al., 2019). Forest succession is accompanied with a shift in species dominance. Pine and oak are the principal and dominant species in subtropical forest of China, which are extremely important both ecologically and economically. However, the mechanisms driving the internal succession of pine-oak forest and its ecosystem functioning are still a debt (Kang et al., 2017; Reyna et al., 2019; Martin et al., 2021; Singh et al., 2021). Soil microorganisms are involved in critical forest ecosystem functions, playing critical roles in forest biogeochemical processes, determining the fate of organic material that enters the soil, driving the carbon flux and nutrient transformation among organisms, resulting in the formation of the forest soil organic matter profiles (Roesch et al., 2007; Islam et al., 2022; Camenzind et al., 2023). On the other hand, tree species also influences the microbial residue and soil organic matter accumulation (Jing et al., 2023). Litter type and quality often have dominated controls on main microbial groups during their decomposition (Bai et al., 2024). Plant–microbe–soil interactions control over decomposition vary widely depending on specific microbial taxa and their functional capabilities, as well as their responses to environmental stressors (Osburn et al., 2022; Badger Hanson and Docherty, 2023; Zhu et al., 2024). Consequently, the adaptive plant–soil interactions shape specific microbial taxa that would determine the succession of plant residue chemistry (Wang et al., 2023).

Plant–microbial interactions in forest floor are increasingly recognized as important drivers of terrestrial ecosystem biogeochemical cycling (Van der Putten et al., 2013). For local microbial community that is relatively stable before critical disturbance, such as the vegetational pine-oak stands replacement, their representative community characteristics are recorded in the original soil where they resided (hereafter, the original soil). Although plant–microbe–soil interactions have been extensively studied (Lambers et al., 2009; Bakker et al., 2014), reciprocal transplantation of original soils in contrasting forest, and detailed investigation of microbial community composition, could be helpful for further understanding of the specificity of field-microbial associations. A theoretical relationship between soil organisms and plant has received much attention in the recent years, which is identified as the home-field advantage effect (HFA) in the litter decomposition (Gholz et al., 2000; Zhao et al., 2024; Zhu et al., 2024). HFA indicates a litter-field affinity effect that litter decomposes faster in an area dominated by the plant species from which it derives (i.e., at home) than in an area dominated by another plant species (i.e., away), and it implies that a decomposer community can become specialized over time to degrade specific litter types they encounter, leading to a quantitative demonstrated advantage for decomposition in the home environment of the litters (Hansen, 1999; Keiser et al., 2011). Meanwhile, local adaptation of microbial community or functional specialization in HFA can be regarded as a type of ‘species fitness difference’-based deterministic process. Traditionally, local community assembly is thought to be determined predominantly by certain environmental conditions, i.e., the idea that stated ‘everything is everywhere but the environment selects’ (Becking, 1934). For a given forest stand, local root status has been demonstrated to be critical affecting the microbially conducted leaf litter decomposition of native plant species (Tian et al., 2018). However, the extent to which the local rhizosphere community is integrated with plant communities (e.g., forest type) has received litter attention probably due to the lack of expertise and methodologies tailored to specific taxa (Montagna et al., 2018). Root exudates can modify the chemical turnover in both litter and soil, and rhizosphere microbial composition would be affected (Chaparro et al., 2014), thus microbial community may be specified in contrasting forest soil. Functional metagenomic analyses have revealed drivers’ functional gene groups due to the presence of specialized rhizosphere niches (Li et al., 2014), and microbial community composition would be expected to be different between the bulk and specific root present in soils (Burns et al., 2015).

Above all, microbial community would adjust its functionality in response to initial species-specific plant–soil interactions (Manzoni et al., 2010). Different vegetation’s can produce heterogeneous soil properties and alter the composition of soil microbial community (Ma et al., 2004; Sundqvist et al., 2011; Mitchell et al., 2012a,b). Increasing efforts have been found to explain microbial community variations by simultaneously considering vegetation composition and soil characteristics (Mitchell et al., 2012a,b). Nevertheless, understanding of how specific forest stand type would affect microbial communities through their cultivation/government of local original soil, and through bio-interactions mediated by belowground root status, is still unclear. In this study, we aim to fill this knowledge gap by exploring the microbial community characteristics in soil samples of a pine-oak forest, determining how it changes after reciprocal transplantation of original soils of different forest stand types to simulate vegetation replacement under nature succession or certain environmental disturbance. We hypothesized that both the incubation field and local root types (tree species and their root present/absent status) would affect the microbial community composition and its variation across the transplantation. Moreover, according to the definition of microbial community resistance and resilience, a community is resistant if its bacterial community composition is not altered after a disturbance, while it is resilient when it recovers after an initial alteration reassembling to the original community composition or a new stable state (Shade et al., 2012; Meola et al., 2014). The ecological stability of native microbial communities is also evaluated, which is available to be calculated as the resistance (after being transplanted away) and resilience (of alien soil recovery toward local microbes) of the microbial community in the pine and oak forest stands.

## 2 Materials and methods

### 2.1 Study site

The reciprocal transplantation experiment was conducted in Nanjing Zijinshan National Forest Park (3008.8 ha., 447.1 m asl, 32°50′N, 118°48′E), Jiangsu province, China. The Zijinshan is an isolated mountain in city Nanjing, and the forest is dominated by a pine species *Pinus massoniana* and an oak species *Quercus* var*iabilis*. Detailed climate, soil, and flora can be found in the study by Lin et al. (2017). In this study, two forest stands were selected, i.e., a broadleaved forest dominated by *Q. variabilis* (QF) and a coniferous forest dominated by *P. massoniana* (PF). Under recent ecological preservation and forest management of the National Forest Park, the natural restoration succession of the core zone tends to filter out *P. massoniana*, and this pioneer pine species would mainly be found in crowds in the intense disturbance areas, such as the arid ridges at high altitude. On the other hand, the climax community would be oak-dominated.

### 2.2 Ingrowth core preparation and soil transplantation

**Figure 1:**
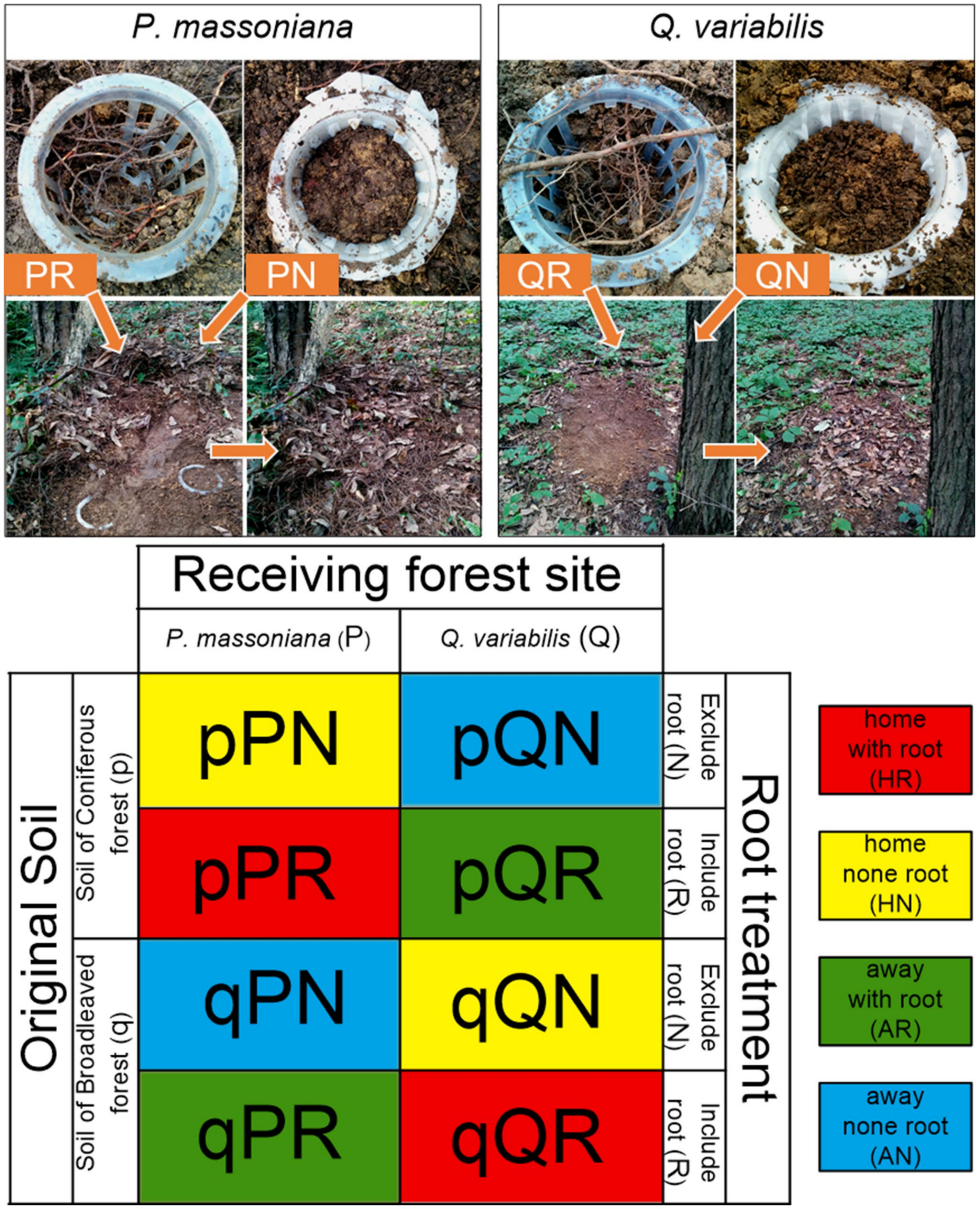
Scheme illustrating the experimental design. The upper panel illustrates the process of belowground ingrowth core incubation; the lower panel illustrates all treatment combinations. The lowercase “p” and “q” represent original soil from the pine and oak forest stand, the capital “P” and “Q” represent receiving incubation forest of PF and QF, the letter “N” and “R” represent treatment excluding and including tree roots in the ingrowth incubation cores.

This study is focused on the biotical association patterns of forest stand type and soil microbes, but not geographical or spatial pattern influences. Consequently, the transplantation was designed to be conducted at two spatially adjacent forest stands in this subtropical theropencedrymion, and the parent soil material was same in the two stands. The difference of microbial composition in contrasting soil samples would mainly be derived from their historical co-adaptation with local vegetation types. The transplant incubation is characterized by three conceptual experimental parameters: the ‘original soil type’ (containing the original microbial community of corresponding forest), the ‘receiving forest’ (the forest stand where soil samples were incubated), and the ‘root status’ (treatments that include or exclude tree roots). The original soil is treated to represent the initial microbial community before vegetational environment changes. Within each forest stand, three dominant trees were selected, the ground layer of which was clearly cut for setting belowground root ingrowth cores. The ingrowth cores were set by using plastic planting baskets with railing barriers (top diameter 10.5 cm, bottom diameter 7.5 cm, and height 7.5 cm), which were buried around the selected trees. Half of the baskets were surrounded with 50 μm nylon mesh to exclude fine roots of the focal tree (root excluding treatment). Before transplantation incubation, soil samples originated from each forest stand were previously homogenized *in situ*; visible root rocks and roots were removed by hand and soils were sieved by 2-mm mesh. The homogenized soils were filled back into the cores at original density as the original soil, i.e., soil from the *P. massoniana* forest (PS) and soil from the *Q. variabilis* forest (QS). Accordingly, the transplantation design can be differentiated as “home” and “away,” as shown in Figure 1. For the root included treatment, fine roots of the selected tree were carefully separated from soil and passed through the basket’s barriers. Then, the homogenized soil was filled into each core and buried with litters around the forest floor (Figure 1). The homogenized original soil samples were transplanted at the two forests at the growing season from 9th July 2017 to 13thJuly 2017 and then incubated for 2 months. Four of the ingrowth cores were destroyed by animals during the incubation, so we harvest 20 effective samples. After the incubation, rhizosphere soil in the root included treatments (fine roots removed), and bulk soil in root excluded treatments, was sieved by 2-mm mesh and stored at −40°C for further analyses.

### 2.3 Soil chemical component, DNA extraction, and amplicon sequencing

Soil element compositions were characterized by using X-ray Fluorescence (XRF, Thermo ARL 9900). Microbial DNA from the soil samples was extracted by using QIAamp Fast DNA Stool Mini Kit (QIAGEN, Germany). The fungal and bacterial community of soil samples were characterized by amplicon sequencing, with internal transcribed spacer (ITS) region primers of ITS1f (5′-CTTGGTCATTTAGAGGAAGTAA-3′) and ITS2r (5′-TCCTCCGCTTATTGATATGC-3′) for fungi and 16S primers 341F (5′-CCTACGGGNGGCWGCAG-3′) and 785R (5′-GACTACHVGGGTATCTAATCC-3′) for bacteria. Samples were sequenced using the Illumina HiSeq 2000 platform (Illumina, America) at Shanghai Genergy Biotechnology Co., Ltd. DNA libraries were prepared following the instructions of Illumina. Cluster generation, template hybridization, isothermal amplification, linearization, blocking, and denaturing and hybridization of the sequencing primers were performed according to the workflow indicated by the provider. Flexbar was used to trim the adapter from the reads (Dodt et al., 2012). The rarefied operational taxonomic unit (OTU) table was generated through QIIME (Caporaso et al., 2010). Consensus sequences were constructed for each cluster, and the OTUs were constructed by clustering these consensus sequences at 97% identity. The raw sequence data for this study are available in the Sequence Read Achieve (SRA) database of the National Centre for Biotechnology Information (NCBI) with BioProject accession number PRJNA1080048.

### 2.4 Data analyses

#### 2.4.1 Microbial community composition

The α-diversity of each sample was determined using the inferred abundance and richness of OTUs. Full factorial ANOVA was applied to test the effect of original soil, receiving forest and root status, as well as their interactions between microbial richness and diversity index. The identified fungal OTUs were further reclassified according to their functional groups by FUNGuild (Nguyen et al., 2016). Compositional differences of microbial communities were assessed to distinguish between common and unique microbes. Indicator species analyses were conducted for bacteria at phylum levels and for fungi at trophic mode, so as to detect the representative species that most associated with certain incubation combinations. The indicator value (*IndVal.g*) was also calculated by using the R package *indicspecies* (De Cáceres and Legendre, 2009; De Cáceres et al., 2010), to identify the indicator species for critical incubations:


Indval.g=Nk∑i∈CaiNiNk∑i∈KaiNi×∑i∈Cni∑i∈CNi

where *N* is the number of samples in the data set, *N_i_* is the number of sample belonging to the treatment *I*, *n* is the number of sample where target species occurs, and *n_i_* is the number of sample in treatment *i* where it occurs, *a* is the sum of abundances of the target species over all samples, and *a_i_* is the sum of its abundances in treatment *i*. Moreover, *K* is the set of all *k* treatment groups and *C* is a set of *c* treatment groups, conforming a particular treatment-group combination.

#### 2.4.2 Microbial associations

Microbial correlations were calculated using Spearman’s rank-order correlation. Positive and negative correlations between microorganisms account for beneficial (mutualism, commensalism, etc.) and detrimental interactions (antagonism, competition, etc.), respectively, permitting a description of the dominant type of interaction among soil microbes. Bilateral co-occurrence molecular ecological networks (MENs) were constructed on the basis of Pearson correlation coefficient, based on random matrix theory (RMT) (Yuan et al., 2021). Nodes in isolation after the threshold of 0.4 (*r* ≥ 0.4 and *p* < 0.05) were retained in the network. Topological indices for weighted networks were calculated in the R package *igraph*.

#### 2.4.3 Microbial community variations

Non-metric multidimensional scaling (NMDS) was employed to evaluate the microbial β-diversity using Bray–Curtis distance. The significance of observed differences of microbial communities among experimental treatments was determined by permutational multivariate analysis of variance (PERMANOVA) with Bray–Curtis dissimilarity after 999 permutations. Constrained ordination analysis was conducted to evaluate the relative contribution of experimental factors in explaining microbial community variations. These analyses were conducted separately for the main species (OTUs with a relative abundance ≥1%) and rare species (OTUs with a relative abundance <1%) by using the R package *vegan*. Distance-based redundancy analysis (db-RDA) was the first choice to test for the experimental effects on microbial community variations among samples in this study, based on microbial relative abundance (RA) data. The db-RDA applies principal coordinate analysis to obtain new Euclidean axes from the RA matrix and fully represent the relationships among samples. In contrast to the commonly used Bray–Curtis distance, the Euclidean method works well in the fine-scale analysis (such as our studying scale). In case the data did not fit a linear analysis, constrained correspondence analysis (CCA) was performed to replace the db-RDA. To evaluate the forest (or soil)-specific ecological stability of the microbial communities, Bray–Curtis dissimilarity matrix between contrasting treatment groups (*D_Bray_*) was calculated representing mean between-group difference of microbial community structure, which could represent the community variations stemmed from transplantation and root exclusion.

According to the soil-vegetation combinations, specific local root status was treated as a single factor, i.e., ‘receiving root’, which contained four types: none root in PF (PN), none root in QF (QN), including root of *P. massoniana* (PR), and root of *Q. variabilis* (QR). All statistic calculations and figures were performed in R version 4.2.2 (R Core Team, 2022).

## 3 Results

### 3.1 Microbial community diversity

**Figure 2:**
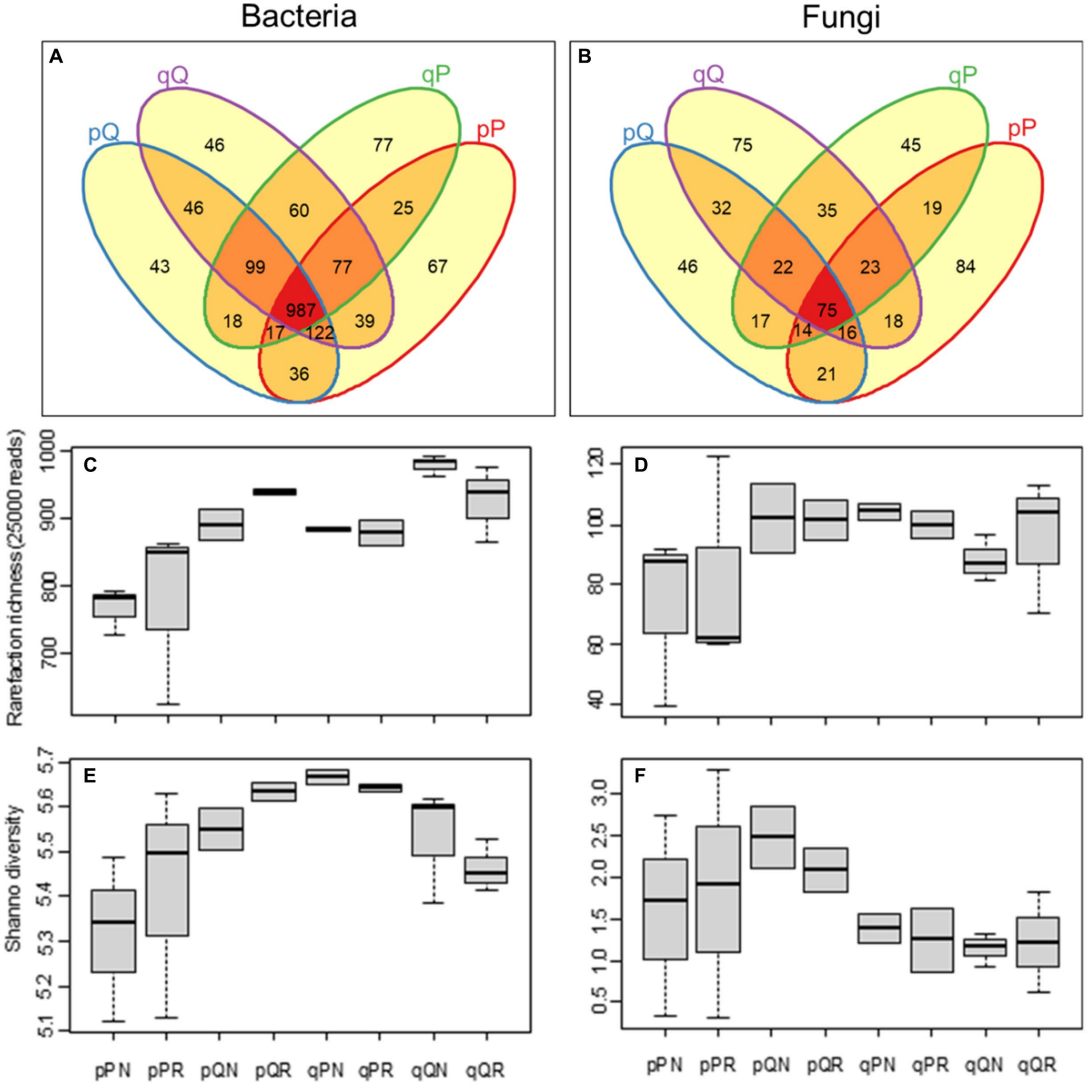
Soil microbial diversity. **(A,B)** Show the Venn diagram of the bacterial and fungal OTU richness among original soil-receiving forest combinations; **(C,D)** show the rarefied bacterial and fungal richness among experimental treatment; **(E,F)** show the Shannon diversity index of the bacteria and fungi communities. The lowercase “p” and “q” represent original soil from the pine and oak forest stand, the capital “P” and “Q” represent receiving incubation forest of PF and QF, the letter “N” and “R” represent treatment excluding and including tree roots in the ingrowth incubation cores.

Based on 41,337 ± 4,715 and 41,883 ± 6,098 reads for bacteria and fungi, an average of 914 ± 101 bacterial and 99 ± 22 fungal OTUs were identified per sample (Supplementary Tables S1, S2). Among original soil-receiving forest interactions, more than half of the bacteria (56.11%) were common to all soil-vegetation combinations, and there was a lower occurrence of specialist bacteria (13.25%) that are specialized for unique type of soil–forest combination (Figure 2A). Contrary to this, there was a lower occurrence of generalist (13.84%) than specialist (46.13%) fungi among soil-forest interactions (Figure 2B). Notably, home incubation had more specialist fungi (84 OTUS for PS soil in PF forest and 75 OTUs for QS in QF) than away incubation (46 and 45 OTUs for PS in QF and QS in PF, respectively).

Plant root had little influence on microbial community diversity (with vs. without root, *p* > 0.05). The PS soil had less bacterial richness than the QS soil (*F* = 8.234, *p* = 0.010), and soil samples incubated in the PF forest had less bacterial richness than in the QF forest (*F* = 17.851, *p* < 0.001, Figure 2C). This suggests that the pine forest may support less bacterial species than the oak forest. Moreover, there was a significant soil–vegetation interactions on the Shannon diversity index of bacteria (*F* = 10.006, *p* = 0.006), with the PS soil samples in the PF forest had lower diversity than in the QF forest, and the QS soil samples in the PF forest had higher diversity than in the QF forest (*F* = 8.428, *p* = 0.013); the bacterial diversity became higher when transplanted away than that incubated at home (Figure 2E). Recognized fungal OTU richness in PS soil was slightly less than in QS soil (*F* = 3.074, *p* = 0.097, Figure 2D), but its Shannon diversity was significantly higher (*F* = 4.466, *p* = 0.049, Figure 2F). This reflected a lower fungal evenness in QS soils (0.264 ± 0.024) than in PS (0.425 ± 0.065; Supplementary Table S2).

### 3.2 Microbial community composition and species association relationship among soil samples

**Figure 3:**
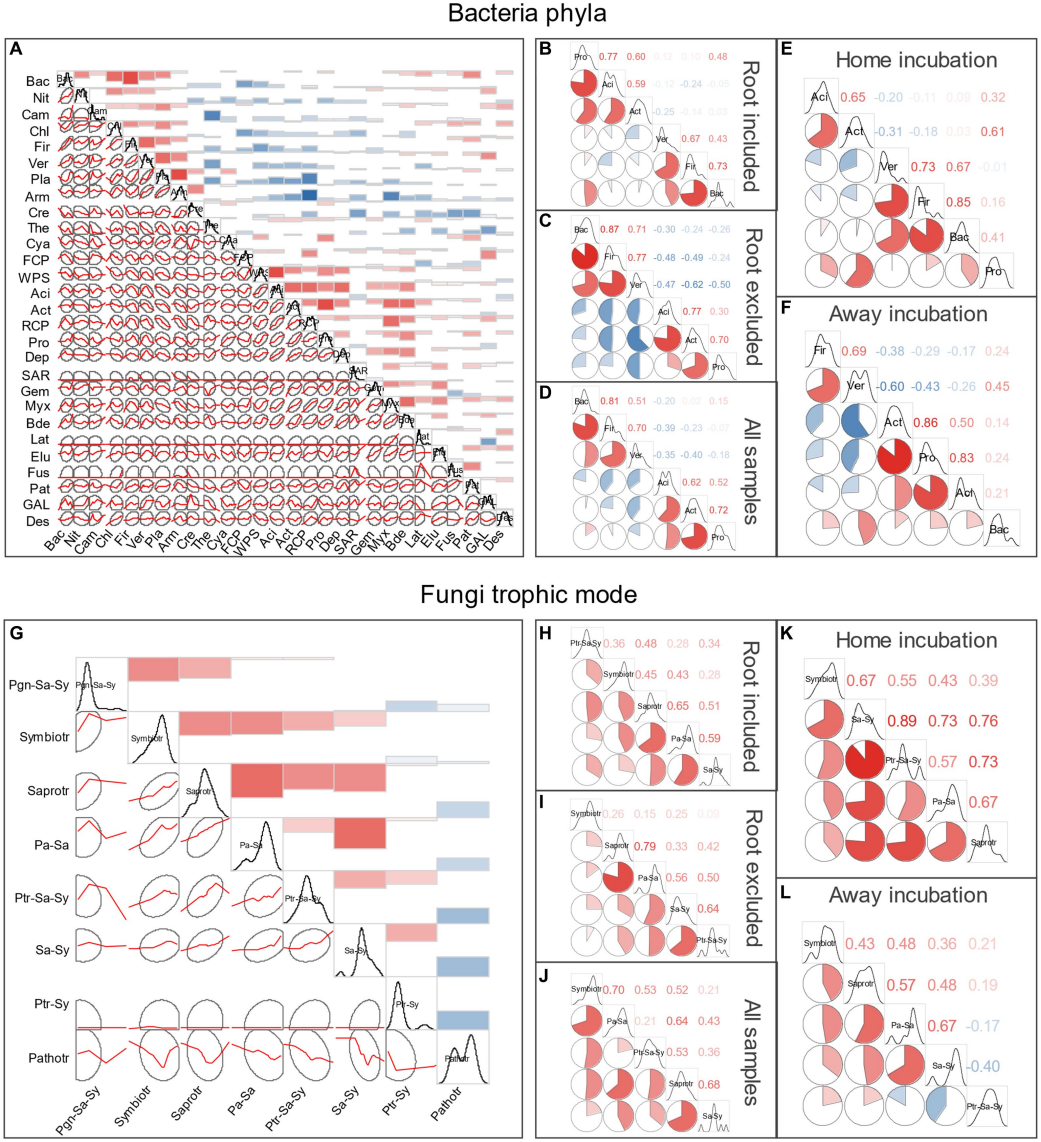
Spearman’s correlations of bacteria taxa at phylum level **(A–F)** and fungi trophic modes according to FUNGuid **(G–L)**. Taxa abbreviations can be found in Abbreviations.

According to the Spearman’s rank-order correlations of bacteria at phylum level, 72.22% of the significant ones (54 pairs in total) were positive, indicating that beneficial associations were adopted (Figure 3A). Generally, two positive sub-groups could be separated, one contained Acidobacteria and Actinobacteria, the first and fifth most abundant phyla, and the other contained the seventh and twelfth most abundant phyla Planctomycetota and Armatimonadota, and they were negatively correlated with most of the other ones. Similar positive association pattern was found for the fungi trophic mode, only the obligate Pathotroph that had negative relations with the others (Figure 3G). Association patterns of the most abundant bacterial phyla (more than 25,000 reads in total) and fungi trophic modes (more than 9,500 reads in total) under different experimental treatments are shown in Figure 3. For bacteria, the six most abundant phyla could be divided into two groups (within-group phyla were positively correlated with each other), one included Acidobacteriota, Actinobacteriota and Proteobacteria, the other group was consisted of Verrucomicrobiota, Firmicutes, and Bacteroidota (Figures 3B–D). Interestingly, such association pattern was intensified when root was excluded, with the between-group phyla became more negatively correlated (Figure 3C), but root could weaken the negative correlations between the two groups (Figure 3B). Similarly, the bacteria correlation pattern became less detrimental when incubated at home than away (Figures 3E,F). For fungi, the five most abundant trophic groups were mainly positively correlated, but some negative relationship appeared when incubated away (the facultative pathotroph–saprotroph–symbiotroph was negatively correlated with the facultative saprotroph–symbiotroph).

**Figure 4:**
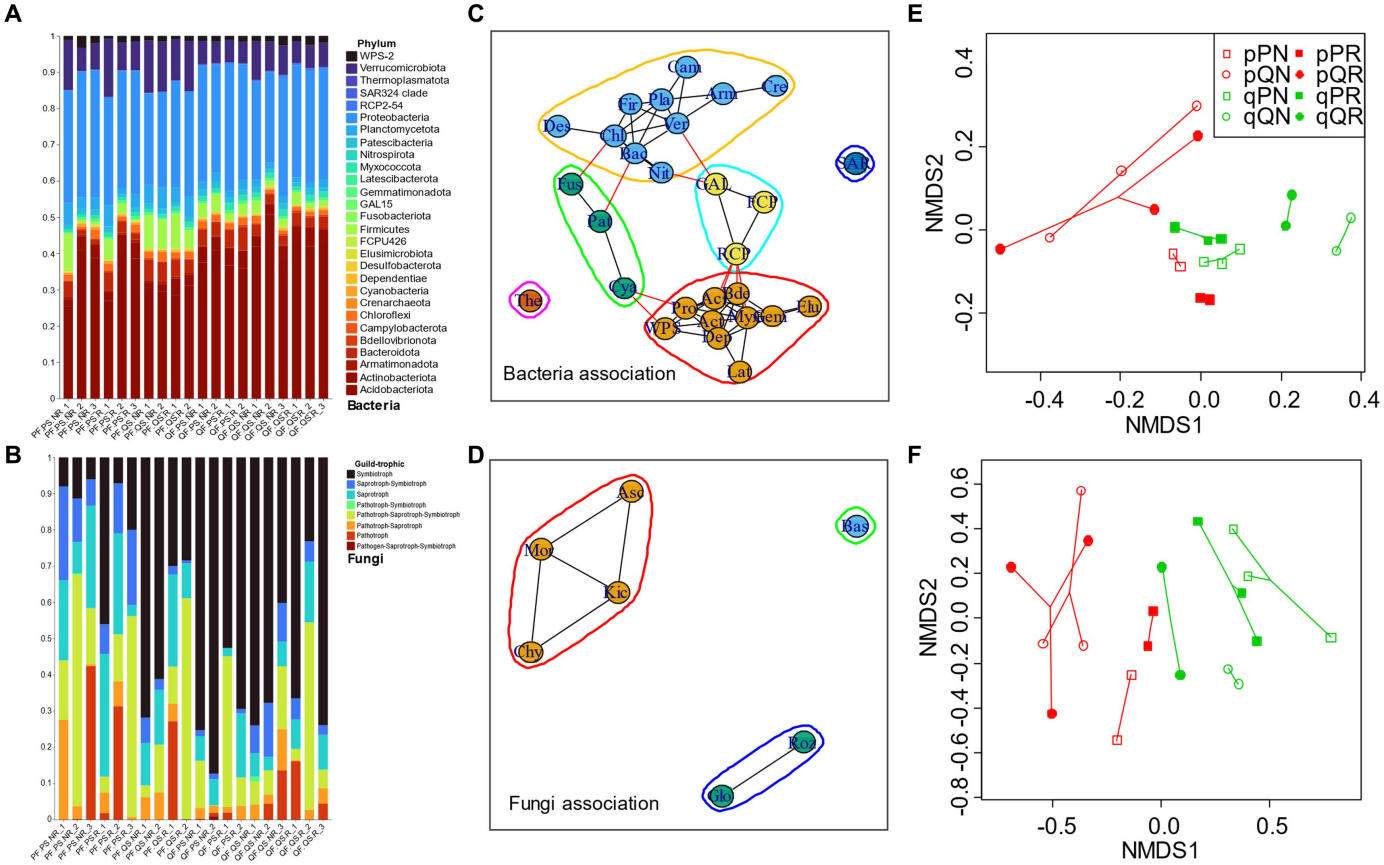
Microbial community composition and beta-diversity. **(A)** Bacterial phyla composition in each soil sample; **(B)** fungal trophic composition in each sample according to FUNGuid; **(C,D)** bacteria and fungi associations at phylum level; **(E,F)** show the beta-diversity pattern of bacteria and fungi by NMDS. The lowercase “p” and “q” represent original soil from the pine and oak forest stand, the capital “P” and “Q” represent receiving incubation forest of PF and QF, and the letter “N” and “R” represent treatment excluding and including tree roots in the ingrowth incubation cores. Taxa abbreviations can be found in Abbreviations.

Most of the fungi OTUs were belonged to the Ascomycota and Basidiomycota (account for 97.42% of the total identified OTU reads, Supplementary Figure S1A), and we analyzed their functional composition by FUNGuid. According to taxonomic classification of bacteria at phylum level and FUNGuid classification of fungi among samples, the compositional variations of bacteria were much less than fungi, with Acidobacteriota and Proteobacteria being the most abundant phyla of the bacterial community in each soil sample (account for 67.58% of the total reads) (Figures 4A,B; Supplementary Figure S1B). Under a threshold of *ρ* > 0.4 on the important positive associations at phylum level, six bacteria modules and three fungi modules were identified (Figures 4C,D). The bacteria consisted of four clusters, the first one consisted of Acidobacteriota, Proteobacteria, Actinobacteriota, etc., and the second one included Verrucomicrobiota, Firmicutes, Armatimonadota, etc. These two large clusters had a minor connection with the other two small ones, i.e., the third cluster (connected the first cluster through Patescibacteria and Fusobacteriota and connected the second cluster with Cyanobacteria) and the fourth cluster (connected the first cluster through RCP2-54 and connected the second cluster with GAL15). The fungal phyla consisted of two clusters (one was consisted of Ascomycota, Mortierellomycota, Kickxellomycota, and Chytridiomycota, and the other one was consisted of Glomeromycota and Rozellomycota) and one individual module with a sole phylum (Basidiomycota, the most abundant taxon), and these three modules did not have any connections with each other. For the fungi trophic mode, the classified symbiotroph (mainly ectomycorrhizal) in the PF forest (especially for the home incubation, i.e., PS in PF) was much lower than in QF forest. On the contrary, the saprotroph was less abundant in QF. Among soil samples, original soil significantly affected bacterial (PERMANOVA *F* = 3.118, *p* = 0.002) and fungal (PERMANOVA *F* = 8.7662, *p* = 0.001) community structures. NMDS1 clearly differentiated original soil for both the bacteria and fungi communities, with the QS cluster higher than PS (Figures 4E,F). Whether including roots or not displayed inconsistent influences on microbial communities. For the bacteria, the receiving forest can also be divided by NMDS2, with larger QF than PS (PERMANOVA *F* = 2.303, *p* = 0.011).

### 3.3 Explanation On microbial community variation

**Figure 5:**
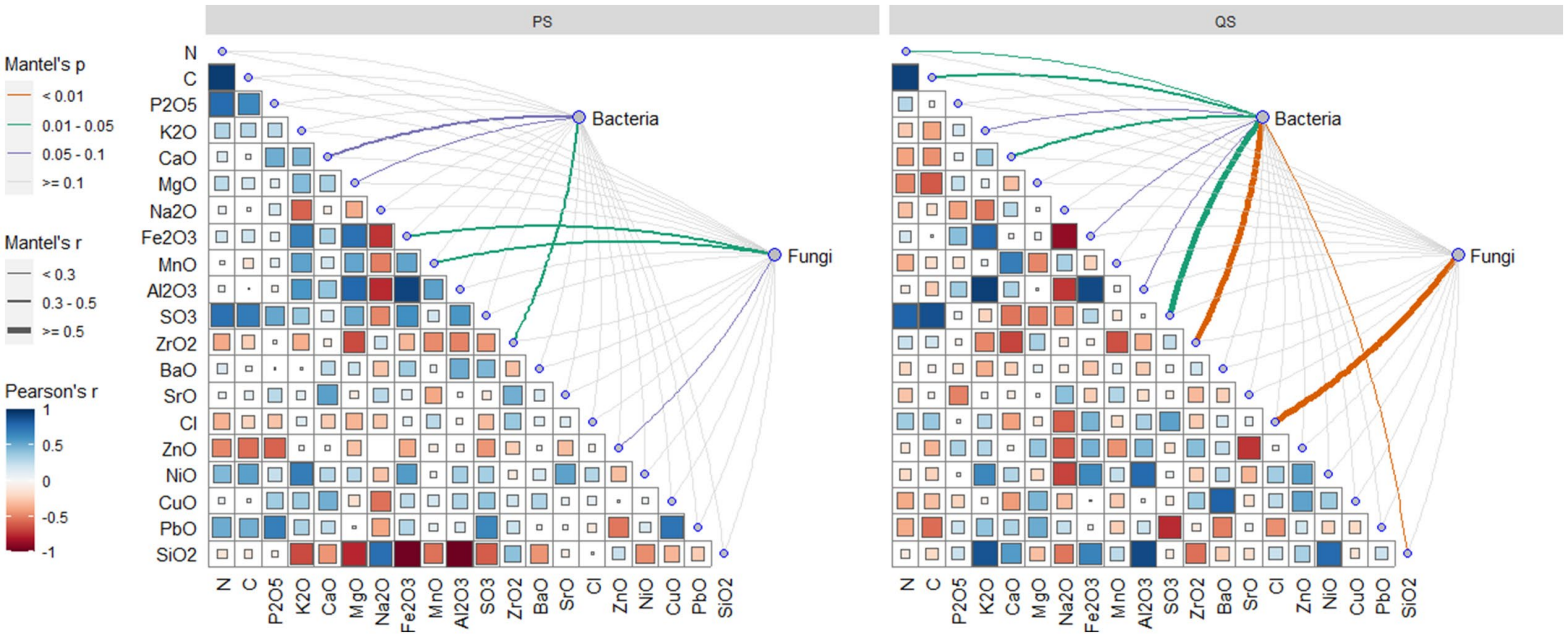
Mantel test of relationships between microbial communities and soil chemical element contents of soil samples belonging to the PS and QS soils.

Soil chemicals were more influential for microbes in the QS soils than in the PS soils. For QS soils, the bacteria were only significantly related to soil Zr, while the fungi were influenced by soil Fe and Mn. For QS soils, the fungi were only influenced by soil Cl, while the bacteria community can be determined by soil C, N, Ca, S, Zr, and Si. Overall, in this study, the Mantel test showed that soil chemicals had limited influence in determining the microbial community, with relatively low Mantel’s r (Figure 5). Accordingly, we did not engage soil chemicals as environmental factors in the constrained ordination models.

The db-RDA and CCA models showed that experimental factors had better explanatory power on community structure of the dominant species than for the rare OTUs, except for the model taking the receiving root as a single predictor, where rare microbes were more depended on soil root types (Tables 1, 2). Original soil alone significantly predicted a large proportion of dominant fungi variations (29.39%, *p* = 0.004) and explained 14.91% of variations of dominant bacteria, but its significant influences on rare microbes were much less. Receiving forest explained 30.87% of the variation of bacterial phylum composition (Supplementary Table S3; *p* = 0.001), but the explanatory power decreased at the OTU level. Whether including or excluding root did not have any significant effect on microbial variations (*p* > 0.1), but specific root types demonstrated an important role in determining microbial community structure, especially for the rare species (accounting for 17.60% of the variations, *p* < 0.05). When the model predictors were original soil plus receiving root types, the explanatory power of constraint variables became largest. Specifically, original soil + receiving root (hereafter, the optimal model) could explain 30.82 and 39.88% of the dominant bacteria and fungi variations among samples while explained 26.44 and 24.31% of the rare bacteria and fungi variations (Tables 1, 2).

**Table 1:** Summary of the db-RDA for effects of experimental effects on soil bacterial composition.

db-RDA on dominant bacteria OTUs					db-RDA on rare bacteria OTUs				
Variation		ANOVA			Variation		ANOVA		
Constrained (explained)	Unconstrained	Predictor	*F*	*P*	Constrained (explained)	Unconstrained	Predictor	*F*	*P*
RA ~ original soil	RA ~ original soil								
14.91%	85.09%	Soil	3.1536	**0.009**	8.41%	91.59%	Soil	1.6533	**0.001**
RA ~ receiving forest	RA ~ receiving forest								
11.06%	88.94%	Forest	2.2395	**0.045**	8.17%	91.83%	Forest	1.6025	**0.001**
RA ~ root inclusion/exclusion treatment	RA ~ root inclusion/exclusion treatment								
2.01%	97.99%	Root	0.3686	0.969	4.55%	95.45%	Root	0.8586	0.934
RA ~ receiving root	RA ~ receiving root								
15.54%	84.46%	Root2	0.9813	0.441	17.60%	82.40%	Root2	1.139	**0.022**
RA ~ original soil + receiving forest	RA ~ original soil + receiving forest								
26.34%	73.66%	Soil	3.4408	**0.005**	17.02%	82.98%	Soil	1.7234	**0.001**
		Forest	2.6388	**0.019**			Forest	1.7634	**0.001**
RA ~ receiving forest + root treatment	RA ~ receiving forest + root treatment								
13.07%	86.93%	Forest	2.1639	**0.048**	12.73%	87.27%	Forest	1.5924	**0.001**
		Root	0.3924	0.956			Root	0.8868	0.867
RA ~ original soil + receiving forest + root treatment	RA ~ original soil + receiving forest + root treatment								
28.35%	71.65%	Soil	3.329	**0.008**	21.57%	78.43%	Soil	1.7162	**0.001**
		Forest	2.5531	**0.028**			Forest	1.7561	**0.001**
		Root	0.4481	0.905			Root	0.9288	0.735
RA ~ original soil + receiving root	RA ~ original soil + receiving root								
30.82%	69.18%	Soil	3.2323	**0.008**	26.44%	73.56%	Soil	1.7154	**0.001**
		Root2	1.1497	0.296			Root2	1.2257	**0.004**

Significant *P* values of ANOVA (*P*<0.05) were presented in bold.

**Table 2:** Summary of the db-RDA and CCA for effects of experimental effects on soil fungi composition.

db-RDA on dominant fungi OTUs					CCA on rare fungi OTUs				
Variation		ANOVA			Variation		ANOVA		
Constrained (explained)	Unconstrained	Predictor	*F*	*P*	Constrained (explained)	Unconstrained	Predictor	*F*	*P*
RA ~ original soil	RA ~ original soil								
29.39%	70.61%	Soil	7.493	**0.004**	7.19%	92.81%	Soil	1.394	**0.001**
RA ~ receiving forest	RA ~ receiving forest								
7.40%	92.60%	Forest	1.438	0.244	6.28%	93.72%	Forest	1.206	**0.003**
RA ~ root inclusion/exclusion treatment	RA ~ root inclusion/exclusion treatment								
3.61%	96.40%	Root	0.673	0.499	5.37%	94.63%	Root	1.021	0.343
RA ~ receiving root	RA ~ receiving root								
13.00%	87.00%	Root2	0.797	0.590	17.60%	82.40%	Root2	1.139	**0.003**
RA ~ original soil + receiving forest	RA ~ original soil + receiving forest								
34.27%	65.73%	Soil	7.602	**0.003**	13.39%	86.61%	Soil	1.411	**0.001**
		Forest	1.263	0.297			Forest	1.218	**0.001**
RA ~ receiving forest + root treatment	RA ~ receiving forest + root treatment								
11.00%	89.00%	Forest	1.413	0.246	11.65%	88.35%	Forest	1.208	**0.006**
		Root	0.689	0.501			Root	1.032	0.265
RA ~ original soil + receiving forest + root treatment	RA ~ original soil + receiving forest + root treatment								
37.88%	62.12%	Soil	7.570	**0.002**	18.76%	81.24%	Soil	1.415	**0.001**
		Forest	1.258	0.282			Forest	1.222	**0.001**
		Root	0.929	0.390			Root	1.057	0.200
RA ~ original soil + receiving root	RA ~ original soil + receiving root								
39.88%	60.12%	Soil	7.334	**0.005**	24.31%	75.69%	Soil	1.424	**0.001**
		Root2	0.872	0.504			Root2	1.131	**0.012**

Significant *P* values of ANOVA (*P*<0.05) were presented in bold.

### 3.4 MEN analyses and indicator species identification

**Figure 6:**
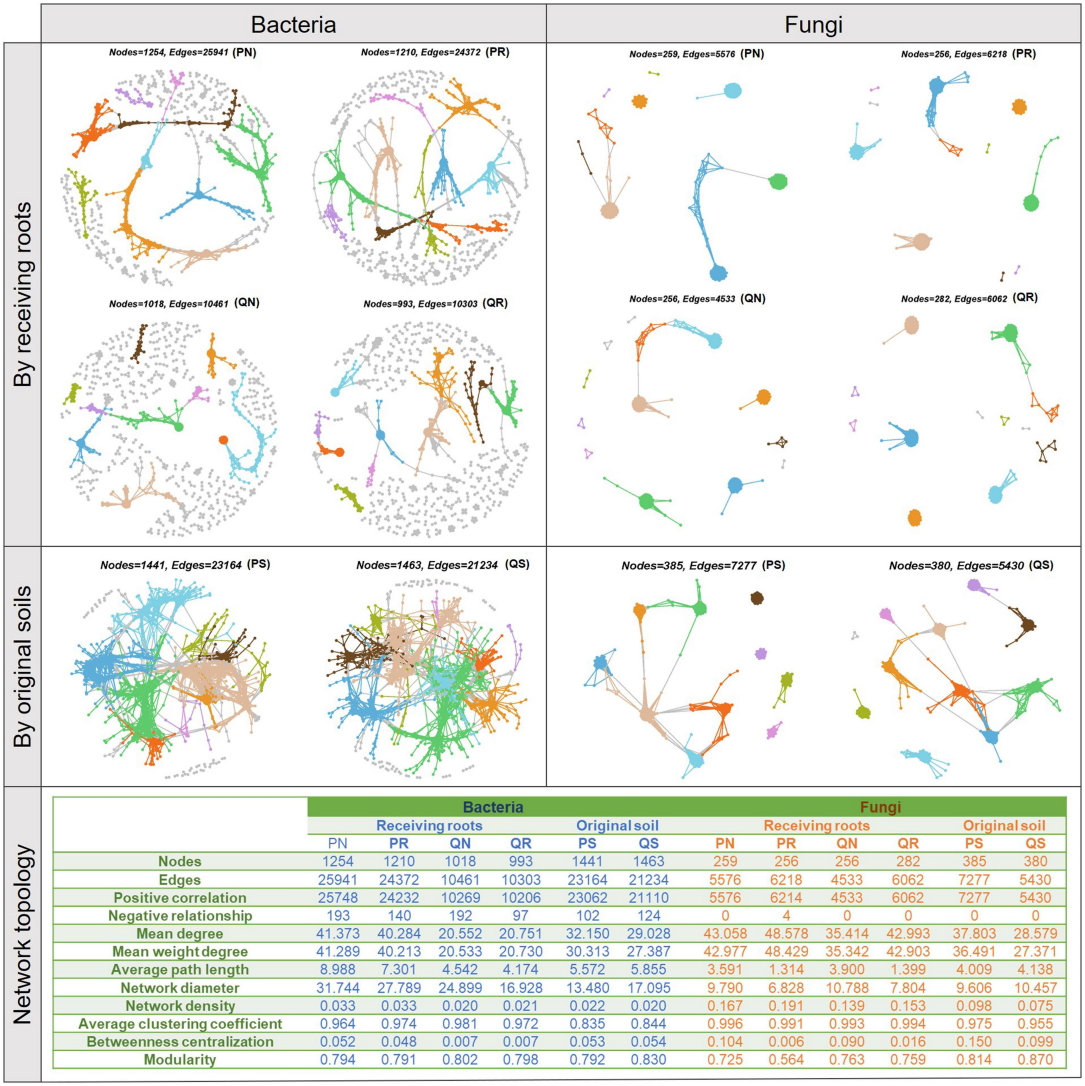
Visualization of constructed MENs based on the original soil and the receiving root.

To explore whether and how experimental factors that engaged in previously selected optimal model would affect ecological relationships within soil microbial communities, MENs were conducted separately for each factor group of the original soil and the receiving root (Figure 6). Network topological parameters showed that the bacteria community network size (total number of nodes), connectivity (total number of edges), density, diameter, the node degree, and the path length were larger in the pine forest than in the soils only growing oak trees, and there were more negative relationships with soil including roots. These indicated a large influence of receiving root types in determining bacterial interactions, while the original soil had little impact on bacteria. For the fungi community, including tree roots increased the network connectivity, density, and node degree while decreased the network diameter, path length, and betweenness centralization. Different from the impact on bacteria, original soil played a prominent influence on fungi community relationships; the coniferous forest soil led to a larger network connectivity, node degree, path length, network density, and betweenness centralization than the oak forest soil, and this indicated that the aboriginal inhabitant types could exert long-term effect on fungi community relationships. In addition, the network modularity of the bacteria communities was similar across experimental groups, while it differed for the fungi communities, with a decrease in modularity when soil containing pine roots. To visualize the among-group differences in the high-order organization of the MENs, main modules were identified. The top 10 modules taken up most of the connections, especially when MENs were separated by original soils. Except for the main modules, bacteria communities in the oak forest contained more small modules than in the pine forest. Indicator species analyses detected 22 bacteria OTUs and 12 fungi OTUs as the indicators of certain receiving root types (Supplementary Tables S4, S5). There were more indicator microbes in the oak forest than in the pine forest, and the number of indicators was slightly increased when roots were excluded.

### 3.5 Ecological succession of microbial community after transplant incubation

**Figure 7:**
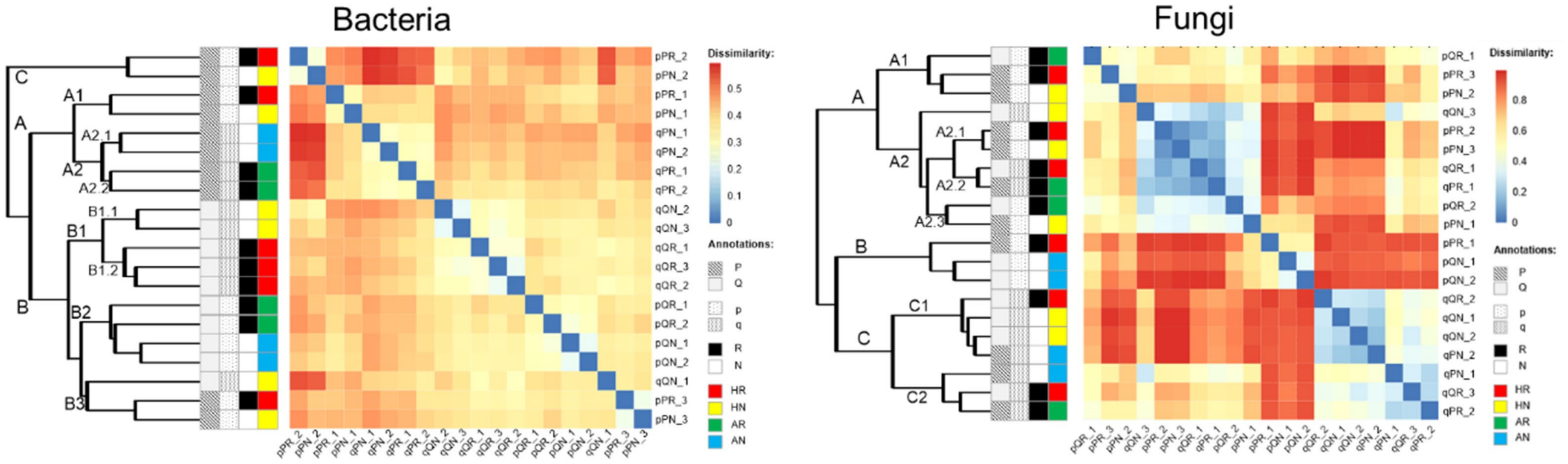
Pairwise comparison of microbial communities among all soil samples and annotations. Clustering and heatmap were computed using the Bray–Curtis dissimilarity. Receiving forest: P, *P. massoniana* forest. Q, *Q. variabilis* forest; original soil: *p*-the PS soil, q-the QS soil; root exclusion treatment: R-root included, N-root excluded. Transplant incubation type: HR-home incubation with root, HN-home incubation without root, AR-away incubation with root, AN-away incubation without root.

Overall, the fungi displayed stronger dissimilarity variations among soil samples than that of the bacteria. The community dissimilarities of bacteria displayed three major clusters (A, B, and C). Clusters A and C were only composed of samples incubated at the PF forest while the cluster B was mainly incubated in the QF forest. Cluster A could further be subdivided into two sub-clusters (A1 and A2). A1 was identical with cluster C, which contained the home incubation PS soils in the pine forest, while A2 composed of the QS soil (away incubation) which could be further subdivided into two sub-clusters according to whether root was excluded (A2.1) or included (A2.2). Cluster B can be subdivided into three sub-clusters (B1–B3). B1 and B2 were incubated only in QF, while B3 also included PF, and all of which were home incubated. B1 only contained QS soil samples for home incubation and could be further divided into two sub-clusters according to root treatment (B1.1: exclude root, B1.2: include root), while B2 only composed of PS soil for away incubation. Similarly, three quantitative clusters could be identified (A, B, and C) but was erratic according to experimental treatments. The cluster A did not contain samples that incubated away without root, and cluster B only contained PS soil, while cluster C only contained QS soil (Figure 7).

**Figure 8:**
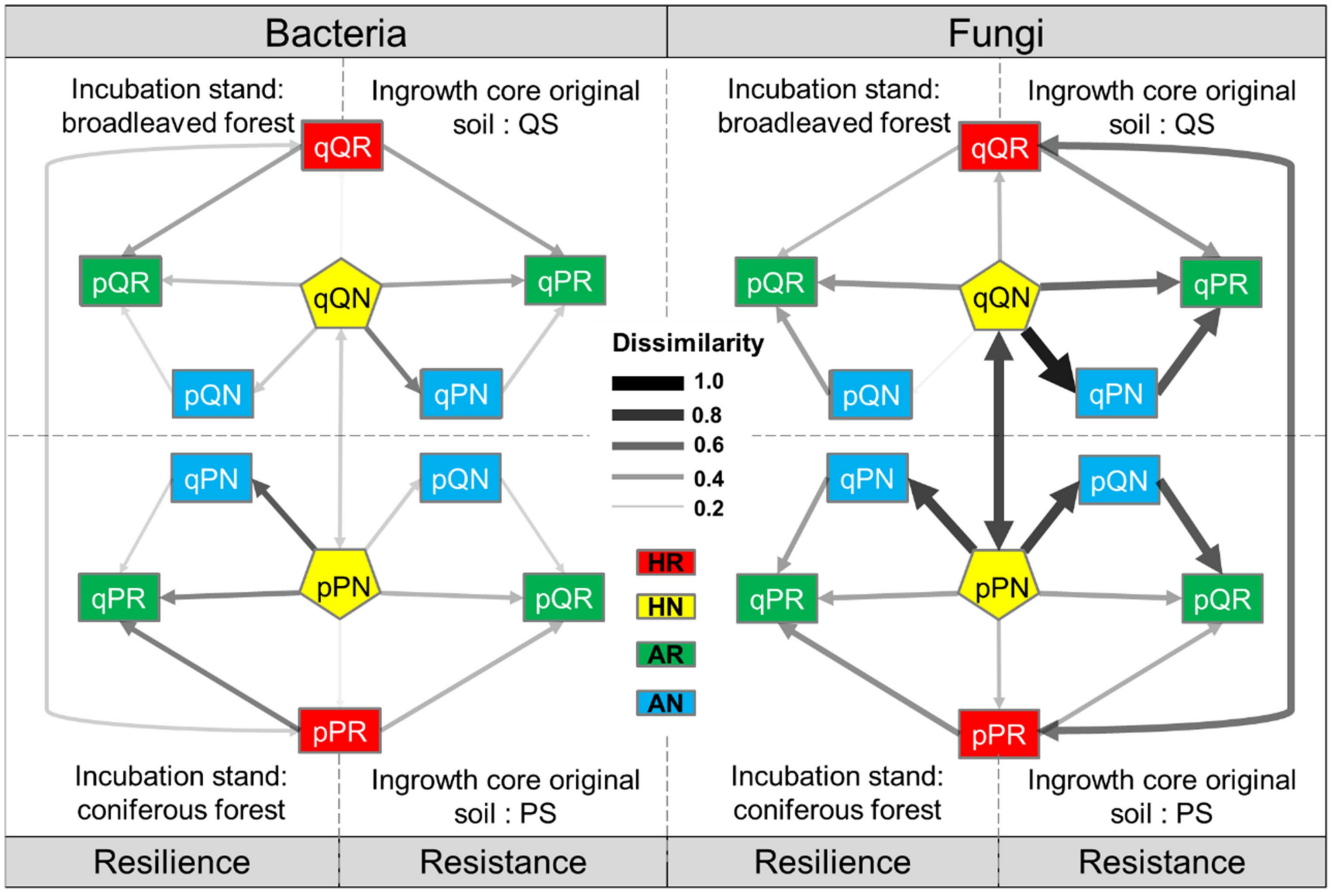
Between-treatment dissimilarity of microbial communities. Transplant incubation type: HR-home incubation with root, HN-home incubation without root, AR-away incubation with root, AN-away incubation without root. Receiving forest: P, *P. massoniana* forest. Q, *Q. variabilis* forest; original soil: *p*-the PS soil, q-the QS soil; root exclusion treatment: R-root included, N-root excluded.

Whether including root or not had weak effect on local (home incubation, red vs. yellow in Figure 8) bacterial community structure (*D_Bray_* = 0.197 for PS in PF, *D_Bray_* = 0.186 for QS in QF), as well as for the away incubation (green vs. blue in Figure 8, *D_Bray_* = 0.226 for PS in QF, *D_Bray_* = 0.246 for QS in PF). This also indicated that the roots of the both tree species had limited influence on soil bacteria. Dissimilarity between the two native incubation (PS in PF, vs. QS in QF) maintained at a stable low value (*D_Bray_* = 0.289 ± 0.006), independent of the root treatment. The QS-originated bacteria displayed a stronger susceptibility (lower resistance) to invasion by extrinsic soil bacteria (*D_Bray_* = 0.335 between away and home incubation with root included, and *D_Bray_* = 0.377 without root) than that of the PS soil (*D_Bray_* = 0.313 with root included, and *D_Bray_* = 0.289 without root). In PF forest, dissimilarity between bacteria of native and alien soils (*D_Bray_* = 0.368 with root and *D_Bray_* = 0.376 without root) was higher than that of QF forest (*D_Bray_* = 0.335 with root and *D_Bray_* = 0.293 without root).

Although root exclusion still had a weak effect on native fungi community structure (*D_Bray_* = 0.266 for PS in PF, *D_Bray_* = 0.268 for QS in QF, Figure 8), it tremendously affected the away incubation in *Q. variabilis* forest (*D_Bray_* = 0.664 for PS in QF) and slightly affected that in *P. massoniana* forest (*D_Bray_* = 0.384 for QS in PF). Contrary to the bacteria, fungi communities were largely differed between the two native incubations and even being depended on root status, (*D_Bray_* = 0.550 ± 0.005 when roots were included and *D_Bray_* = 0.721 ± 0.002 when roots were excluded). The PS originated fungi had a lower resistance to invasion by extrinsic soil fungi than that of QS soil, especially when roots were excluded (*D_Bray_* = 0.730 for PS in QF, and *D_Bray_* = 0.124 for QS in PF). This indicated that fungi had much more resistance capacity. On the other hand, after the reciprocal transplantation, the resilience of native fungi (i.e., fungi community succession of alien soil sample toward the surrounding native forest) was low under the root exclusion treatment, especially for the QF forest (*D_Bray_* = 0.891 for QF, and *D_Bray_* = 0.737 for PF).

## 4 Discussion

Previous study has shown that there was an overall positive litter-field affinity of the two dominate tree species in this subtropical forest (Lin et al., 2017; Tian et al., 2018). The microbial community was supposed to be specified in contrasting forest stands. What determines microbial community structure is key for our understanding of litter-field affinity in determining forest biogeochemical cycles. The origin of the optimized soil community should stem from the differential metabolic capacity of the decomposers and competition in the soil or litter environment (Wickings et al., 2012). In this study, local microbial community variations among soil samples after transplantation, as well as its ecological succession under the influence of plant root types, were resolved.

Different biomes displayed contrasting patterns of microbial composition among the soil-vegetation groups, with bacteria that had a large proportion of generalist OTUs and less specialist, while fungi contained a small proportion of generalist and more specialist. This may be caused by the fact that bacteria are individually short living and extensively dispersed. In this study, the transplantation would actually bring a disturbance, after which stochastic processes would be more pronounced at the beginning of local microbes of the receiving forest colonizing the soil samples and consequently promoting broader niche opportunities and low competition for the *r*-strategist bacteria (Leibold and McPeek, 2006). The fungi, on the contrary (mostly *K*-strategists), were mainly dependent on the long-time formed deterministic processes such as habitat filtering and competition and would become composed of species toward locally adapted taxa (Banning et al., 2011; Ferrenberg et al., 2013).

Higher bacteria richness was found in habitat with *Q. variabilis* (QS and QF) than *P. massoniana* (PS and PF), but the α-diversity was higher when soil samples were transplanted away, despite of the inherent divergence between original soils or receiving forests (Figure 2). For the bacterial community, despite predominate beneficial associations at phylum level (72.22% of all significant pairwise comparisons), some abundant phyla (e.g., Acidobacteriota) displayed a detrimental association with some others, and such detrimental relationships became more predominate for the home incubations. These findings reflect that competition exclusion might be more intense at local incubation, and the disturbance of transplanting away would generate higher bacterial diversity. On the other hand, the diversity of fungi was mainly dependent on original soil, with a much lower evenness in soil samples originated from the oak forest. Studies have demonstrated that the deterministic processes on microbial community assembly could account for only part of the compositional variation, and the stochastic processes often occupied a large portion (Ramette and Tiedje, 2007; Ge et al., 2008; Peay et al., 2010; Zhang et al., 2016). In contrast to deterministic processes, stochastic processes would shape community composition to be functionally equivalent (Hubbell, 2005). Moreover, even the between-group variation of fungi functional composition denoted significant deterministic processes (Figures 2, 4), their large within-group variations may also reflect the critical effect of the stochastic processes.

We found that the root exclusion treatment had weak influence on microbial community structure, but receiving root type was a meaningful explanatory factor, with a species-specific effect of plant root on microbial clusters. When incubated away, root did not affect the microbial clusters among experimental treatments, but for the home incubations, *P. massoniana* root led to lower NMDS1 (bacterial phyla higher in QF and lower in PF, positively correlated with Verrucomicrobiota and negatively correlated with Acidobacteriota), while *Q. variabilis* root led to higher NMDS1. Meanwhile, for the most abundant microbial taxa, excluding root would cause some detrimental associations, e.g., Verrucomicrobiota and Firmicutes versus Acidobacteriota and Actinobacteriota, indicating that plant root would help to sustain the coexistence of dominant microbes. Although exact specific mechanisms underlying MEN associations are unknown with correlation-based network analyses, these associations (correlations between nodes) may in fact indicate certain biological interactions: the positive ones could represent cooperative behaviors (e.g., syntrophic interactions, mutualistic interactions, commensalism, and shared environmental requirements), while the negative ones could reflect detrimental behaviors (e.g., competition for limiting resources, distinctive environmental niches, and spatial isolation) (Yuan et al., 2021). As the original soil keeps historical community assembly, it is a pivotal factor determining microbial community structure in this study, especially for the fungi. Initial colonizers can exclude later-arriving species, a mechanism known as priority effects, as a result of strong interspecific interactions and habitat modification (Alford and Wilbur, 1985; Belyea and Lancaster, 1999; Vannette and Fukami, 2014). The magnitude of priority effects appears dependent on individual species and environmental conditions (Chase, 2007; Kardol et al., 2013; Tucker and Fukami, 2014; Hiscox et al., 2015). In this study, original soil accounted for a large proportion of the dominant fungi regardless of where the samples were incubated (Table 2; Figure 6), reflecting a competitive outcome of the priority effect (or high resistance of the dominate fungi to be replaced). For the large compositional proportion of rare OTUs, corresponding receiving root type became influential on the coexistence of rare species.

There was an interactive effect of transplantation and root status on microbial community successions among samples. Overall, the variation pattern of microbial community in response to the transplantation was different between bacteria and fungi. The QS soil bacteria had higher resistance than PS soil for away incubation; the native and away incubations generated higher dissimilarity of bacterial community in PF forest, and these indicated more stable connections between *P. massoniana* and its native bacteria in PF forest. However, fungal community successions were mostly elusive. For instance, the *Q. variabilis* root status affected the fungi dissimilarity between the two native incubations, the fungi in PS soil had lower resistance than in QS soil, and resilience of local fungi in QF forest was lower with root than without. These findings reflected that the root of *Q. variabilis* could constrain the infection of native fungi (in QF) on abiotic soil substrate (fungi community from PS).

Root exclusion treatment displayed weak influence on ecological succession of bacterial community, while it extensively affected the fungi succession of away incubation (Figure 8). In addition to saprotroph (including dung, leaf, wood, and soil saprotrophs), soil fungi also composed of symbiotroph (mainly ectomycorrhizal in this study) and pathotroph (animal and plant pathogens, fungal, and lichen parasites). Studies focus on the relevance between local microbial community on forest biogeochemical cycles, and the potential roles that root played in regulating this plant–soil interaction should be further refined to explore more specific ways through which specific microbial function groups and their interaction would affect the decomposition of various plant litters. Furthermore, this study is conducted during the peak growing season of the plant community (summer, both the incubation and at the sampling time); we would argue that the data are likely to provide a representative comparison in this bioclimatic zone. Various studies demonstrate that seasonal changes, particularly in soil chemical characteristics, have extensive impacts on rhizosphere microbial communities (Lauber et al., 2013; Regan et al., 2014; Francioli et al., 2018). Thus, while it is possible that seasonal changes in rhizosphere communities may occur and were not detected in this study, it can nonetheless address hypotheses of common diversity patterns. Meanwhile, some other studies indicate that soil microbial assemblages are primarily mediated by stochastic processes (Stegen et al., 2012; Zhang et al., 2016); a given microbial community is portrayed/as chaotic and unpredictable due to its complex, non-linear, and rapidly evolving characteristics (Faust and Raes, 2012). For further exploration, temporal dynamic of microbial succession would be more helpful for detecting the ecological stability of site-specific microbial community and should be conducted in future study.

The differential reaction and structuring of bacteria versus fungi communities suggest that the dominant trees could govern soil processes via root affecting microbial profiling, and the reciprocal transplanted soil microbiome has the potential to expand our understanding of ecological succession and stability of microbial community developed during the forest stand evolution. Overall, local bacteria were more dependent on the place that they were incubated, while fungi were more dependent on priority effects (succession of the original habitat). Between-group variations showed species-specific influences on both bacteria and fungi, and more conservative relationship between *P. massoniana* and its native bacteria in PF forest was found. Root of *Q. variabilis* could constrain the infection of native fungi (in QF) on abiotic soil substrate (fungi community from PS). Among the first *in situ* explorations on potential influence of transplanted soil matrices (microbes) and specific plant root status, the new findings of this study would contribute to our further understanding of the mechanism of local soil microbial community assembly and its functions engaged in the forest biogeochemical cycles.

## Data availability statement

The datasets presented in this study can be found in online repositories. The names of the repository/repositories and accession number(s) can be found in the article/Supplementary material.

## Author contributions

KT: Writing-original draft, Conceptualization, Writing–review & editing, Data curation, Formal analysis, Investigation, Methodology, Validation, Funding acquisition. SC: Writing-original draft, Validation. RY: Writing–review & editing, Methodology, Investigation. YX: Writing–review & editing, Data curation, Formal Analysis. LY: Writing–review & editing, Supervision, Funding acquisition. HL: Writing–review & editing, Conceptualization, Funding acquisition, Resources.

## Figures and Tables

**Table:** 

Bacteria		Fungi	
Phylum	Abbreviation	Trophic mode Phylum	Abbreviation
Acidobacteriota	Aci	Pathogen-Saprotroph-Symbiotroph	Pgn-Sa-Sy
Actinobacteriota	Act		
Armatimonadota	Arm		
Bacteroidota	Bac	Symbiotroph	Symbiotr
Bdellovibrionota	Bde	Saprotroph	Saprotr
Campylobacterota	Cam	Pathotroph-Saprotroph	Pa-Sa
Chloroflexi	Chl		
Crenarchaeota	Cre	Pathotroph-Saprotroph-Symbiotroph	Ptr-Sa-Sy
Cyanobacteria	Cya		
Dependentiae	Dep		
Desulfobacterota	Des	Saprotroph-Symbiotroph	Sa-Sy
Elusimicrobiota	Elu		
FCPU426	FCP	Pathotroph-Symbiotroph	Ptr-Sy
Firmicutes	Fir		
Fusobacteriota	Fus	Pathotroph	Pathotr
GAL15	GAL		
Gemmatimonadota	Gem		
Latescibacterota	Lat	Ascomycota	Asc
Myxococcota	Myx	Basidiomycota	Bas
Nitrospirota	Nitr	Chytridiomycota	Chy
Patescibacteria	Pat	Glomeromycota	Glo
Planctomycetota	Pla	Kickxellomycota	Kic
Proteobacteria	Pro	Mortierellomycota	Mor
RCP2-54	RCP	Rozellomycota	Roz
SAR324 clade	SAR		
Thermoplasmatota	The		
Verrucomicrobiota	Ver		
WPS-2	WPS		

## References

[ref1] Alford R. A. Wilbur H. M. (1985). Priority effects in experimental pond communities: responses of Hyla to Bufo and Rana. Ecology 66, 1106–1114. doi: 10.2307/1939162

[ref2] Anderson-Teixeira K. J. Miller A. D. Mohan J. E. Hudiburg T. W. Duval B. D. DeLucia E. H. (2013). Altered dynamics of forest recovery under a changing climate. Glob. Chang. Biol. 19, 2001–2021. doi: 10.1111/gcb.12194, PMID: 23529980

[ref3] Badger Hanson E. Docherty K. M. (2023). Mini-review: current and future perspectives on Microbially focused restoration strategies in tallgrass prairies. Microb. Ecol. 85, 1087–1097. doi: 10.1007/s00248-022-02150-1, PMID: 36449026

[ref4] Bai X. Zhai G. Wang B. An S. Liu J. Xue Z.. (2024). Litter quality controls the contribution of microbial carbon to main microbial groups and soil organic carbon during its decomposition. Biol. Fertil. Soils 60, 167–181. doi: 10.1007/s00374-023-01792-8

[ref5] Bakker M. G. Schlatter D. C. Otto-Hanson L. Kinkel L. L. (2014). Diffuse symbioses: roles of plant-plant, plant-microbe and microbe-microbe interactions in structuring the soil microbiome. Mol. Ecol. 23, 1571–1583. doi: 10.1111/mec.12571, PMID: 24148029

[ref6] Banning N. C. Gleeson D. B. Grigg A. H. Grant C. D. Andersen G. L. Brodie E. L.. (2011). Soil microbial community successional patterns during Forest ecosystem restoration. Appl. Environ. Microb. 77, 6158–6164. doi: 10.1128/Aem.00764-11, PMID: 21724890 PMC3165429

[ref7] Becking L. G. M. (1934). Geobiologie of inleiding tot de milieukunde. The Hague: W. P. Van Stockum and Zoon, 18–19.

[ref8] Belyea L. R. Lancaster J. (1999). Assembly rules within a contingent ecology. Oikos 86, 402–416. doi: 10.2307/3546646

[ref9] Burns J. H. Anacker B. L. Strauss S. Y. Burke D. J. (2015). Soil microbial community variation correlates most strongly with plant species identity, followed by soil chemistry, spatial location and plant genus. Aob. Plants 7:plv030. doi: 10.1093/aobpla/plv030, PMID: 25818073 PMC4417136

[ref10] Camenzind T. Mason-Jones K. Mansour I. Rillig M. C. Lehmann J. (2023). Formation of necromass-derived soil organic carbon determined by microbial death pathways. Nat. Geosci. 16, 115–122. doi: 10.1038/s41561-022-01100-3

[ref11] Caporaso J. G. Kuczynski J. Stombaugh J. Bittinger K. Bushman F. D. Costello E. K.. (2010). QIIME allows analysis of high-throughput community sequencing data. Nat. Methods 7, 335–336. doi: 10.1038/nmeth.f.303, PMID: 20383131 PMC3156573

[ref12] Chaparro J. M. Badri D. V. Vivanco J. M. (2014). Rhizosphere microbiome assemblage is affected by plant development. ISME J. 8, 790–803. doi: 10.1038/ismej.2013.196, PMID: 24196324 PMC3960538

[ref13] Chase J. M. (2007). Drought mediates the importance of stochastic community assembly. Proc. Natl. Acad. Sci. U. S. A. 104, 17430–17434. doi: 10.1073/pnas.0704350104, PMID: 17942690 PMC2077273

[ref14] De Cáceres M. Legendre P. (2009). Associations between species and groups of sites: indices and statistical inference. Ecology 90, 3566–3574. doi: 10.1890/08-1823.1, PMID: 20120823

[ref15] De Cáceres M. Legendre P. Moretti M. (2010). Improving indicator species analysis by combining groups of sites. Oikos 119, 1674–1684. doi: 10.1111/j.1600-0706.2010.18334.x

[ref16] Dodt M. Roehr J. T. Ahmed R. Dieterich C. (2012). FLEXBAR—flexible barcode and adapter processing for next-generation sequencing platforms. Biology 1, 895–905. doi: 10.3390/biology1030895, PMID: 24832523 PMC4009805

[ref17] Faust K. Raes J. (2012). Microbial interactions: from networks to models. Nat. Rev. Microbiol. 10, 538–550. doi: 10.1038/nrmicro2832 22796884

[ref18] Ferrenberg S. O'Neill S. P. Knelman J. E. Todd B. Duggan S. Duggan D.. (2013). Changes in assembly processes in soil bacterial communities following a wildfire disturbance. ISME J. 7, 1102–1111. doi: 10.1038/ismej.2013.11, PMID: 23407312 PMC3660671

[ref19] Francioli D. Schulz E. Buscot F. Reitz T. (2018). Dynamics of soil bacterial communities over a vegetation season relate to both soil nutrient status and plant growth phenology. Microb. Ecol. 75, 216–227. doi: 10.1007/s00248-017-1012-0, PMID: 28712045

[ref20] Ge Y. He J. Z. Zhu Y. G. Zhang J. B. Xu Z. Zhang L. M.. (2008). Differences in soil bacterial diversity: driven by contemporary disturbances or historical contingencies? ISME J. 2, 254–264. doi: 10.1038/ismej.2008.2 18239609

[ref21] Gholz H. L. Wedin D. A. Smitherman S. M. Harmon M. E. Parton W. J. (2000). Long-term dynamics of pine and hardwood litter in contrasting environments: toward a global model of decomposition. Glob. Chang. Biol. 6, 751–765. doi: 10.1046/j.1365-2486.2000.00349.x

[ref9001] Hansen R. A. (1999). Red oak litter promotes a microarthropod functional group that accelerates its decomposition. Plant Soil 209, 37–45. doi: 10.1023/A:1004506414711

[ref22] Hiscox J. Savoury M. Muller C. T. Lindahl B. D. Rogers H. J. Boddy L. (2015). Priority effects during fungal community establishment in beech wood. ISME J. 9, 2246–2260. doi: 10.1038/ismej.2015.38, PMID: 25798754 PMC4579477

[ref23] Hubbell S. P. (2005). Neutral theory in community ecology and the hypothesis of functional equivalence. Funct. Ecol. 19, 166–172. doi: 10.1111/j.0269-8463.2005.00965.x

[ref24] Islam M. R. Singh B. Dijkstra F. A. (2022). Stabilisation of soil organic matter: interactions between clay and microbes. Biogeochemistry 160, 145–158. doi: 10.1007/s10533-022-00956-2

[ref25] Jing Y. Zhao X. Liu S. Tian P. Sun Z. Chen L.. (2023). Influence of tree species on soil microbial residue accumulation and distribution among soil aggregates in subtropical plantations of China. Ecol. Process. 12:32. doi: 10.1186/s13717-023-00444-x

[ref26] Kang H. Zheng Y. Liu S. Chai Z. Chang M. Hu W.. (2017). Population structure and spatial pattern of predominant tree species in a pine–oak mosaic mixed forest in the Qinling Mountains. China. J. Plant Interact. 12, 78–86. doi: 10.1080/17429145.2017.1283069

[ref27] Kardol P. Souza L. Classen A. T. (2013). Resource availability mediates the importance of priority effects in plant community assembly and ecosystem function. Oikos 122, 84–94. doi: 10.1111/j.1600-0706.2012.20546.x

[ref9002] Keiser A. D. Strickland M. S. Fierer N. Bradford M. A. (2011). The effect of resource history on the functioning of soil microbial communities is maintained across time. Biogeosciences 8, 1477–1486. doi: 10.5194/bg-8-1477-2011

[ref28] Lambers H. Mougel C. Jaillard B. Hinsinger P. (2009). Plant-microbe-soil interactions in the rhizosphere: an evolutionary perspective. Plant Soil 321, 83–115. doi: 10.1007/s11104-009-0042-x

[ref29] Lauber C. L. Ramirez K. S. Aanderud Z. Lennon J. Fierer N. (2013). Temporal variability in soil microbial communities across land-use types. ISME J. 7, 1641–1650. doi: 10.1038/ismej.2013.50, PMID: 23552625 PMC3721119

[ref30] Leibold M. A. McPeek M. A. (2006). Coexistence of the niche and neutral perspectives in community ecology. Ecology 87, 1399–1410. doi: 10.1890/0012-9658(2006)87[1399:Cotnan]2.0.Co;2, PMID: 16869414

[ref31] Li X. Rui J. Xiong J. Li J. He Z. Zhou J.. (2014). Functional potential of soil microbial communities in the maize rhizosphere. PLoS One 9:e112609. doi: 10.1371/journal.pone.0112609, PMID: 25383887 PMC4226563

[ref32] Lin H. He Z. Hao J. Tian K. Jia X. Kong X.. (2017). Effect of N addition on home-field advantage of litter decomposition in subtropical forests. Forest Ecol. Manag. 398, 216–225. doi: 10.1016/j.foreco.2017.05.015

[ref33] Ma X. Chen T. Zhang G. Wang R. (2004). Microbial community structure along an altitude gradient in three different localities. Folia Microbiol. 49, 105–111. doi: 10.1007/Bf02931382, PMID: 15227779

[ref34] Manzoni S. Trofymow J. A. Jackson R. B. Porporato A. (2010). Stoichiometric controls on carbon, nitrogen, and phosphorus dynamics in decomposing litter. Ecol. Monogr. 80, 89–106. doi: 10.1890/09-0179.1

[ref35] Martin M. P. Peters C. M. Asbjornsen H. Ashton M. S. (2021). Diversity and niche differentiation of a mixed pine–oak forest in the sierra Norte, Oaxaca, Mexico. Ecosphere 12:e03475. doi: 10.1002/ecs2.3475

[ref36] Meola M. Lazzaro A. Zeyer J. (2014). Diversity, resistance and resilience of the bacterial communities at two alpine glacier forefields after a reciprocal soil transplantation. Environ. Microbiol. 16, 1918–1934. doi: 10.1111/1462-2920.12435, PMID: 24571618

[ref37] Mitchell R. J. Hester A. J. Campbell C. D. Chapman S. J. Cameron C. M. Hewison R. L.. (2012a). Explaining the variation in the soil microbial community: do vegetation composition and soil chemistry explain the same or different parts of the microbial variation? Plant Soil 351, 355–362. doi: 10.1007/s11104-011-0968-7

[ref38] Mitchell R. J. Keith A. M. Potts J. M. Ross J. Reid E. Dawson L. A. (2012b). Overstory and understory vegetation interact to alter soil community composition and activity. Plant Soil 352, 65–84. doi: 10.1007/s11104-011-0980-y

[ref39] Montagna M. Berruti A. Bianciotto V. Cremonesi P. Giannico R. Gusmeroli F.. (2018). Differential biodiversity responses between kingdoms (plants, fungi, bacteria and metazoa) along an alpine succession gradient. Mol. Ecol. 27, 3671–3685. doi: 10.1111/mec.14817, PMID: 30146795

[ref40] Nguyen N. H. Song Z. Bates S. T. Branco S. Tedersoo L. Menke J.. (2016). FUNGuild: an open annotation tool for parsing fungal community datasets by ecological guild. Fungal Ecol. 20, 241–248. doi: 10.1016/j.funeco.2015.06.006

[ref41] Osburn E. D. Hoch P. J. Lucas J. M. McBride S. G. Strickland M. S. (2022). Evaluating the roles of microbial functional breadth and home-field advantage in leaf litter decomposition. Funct. Ecol. 36, 1258–1267. doi: 10.1111/1365-2435.14026

[ref42] Peay K. G. Garbelotto M. Bruns T. D. (2010). Evidence of dispersal limitation in soil microorganisms: isolation reduces species richness on mycorrhizal tree islands. Ecology 91, 3631–3640. doi: 10.1890/09-2237.1, PMID: 21302834

[ref43] Ramette A. Tiedje J. M. (2007). Multiscale responses of microbial life to spatial distance and environmental heterogeneity in a patchy ecosystem. P. Natl. Acad. Sci. U. S. A. 104, 2761–2766. doi: 10.1073/pnas.0610671104, PMID: 17296935 PMC1815255

[ref9003] R Core Team. (2022). R: A language and environment for statistical computing. Vienna, Austria: R Foundation for Statistical Computing. Available at: https://www.R-project.org/

[ref44] Regan K. M. Nunan N. Boeddinghaus R. S. Baumgartne V. Berner D. Berner S.. (2014). Seasonal controls on grassland microbial biogeography: are they governed by plants, abiotic properties or both? Soil biol. Biochemist 71, 21–30. doi: 10.1016/j.soilbio.2013.12.024

[ref45] Reyna T. A. Martínez-Vilalta J. Retana J. (2019). Regeneration patterns in Mexican pine-oak forests. For. Ecosyst. 6:50. doi: 10.1186/s40663-019-0209-8

[ref46] Roesch L. F. Fulthorpe R. R. Riva A. Casella G. Hadwin A. K. M. Hadwin A. D.. (2007). Pyrosequencing enumerates and contrasts soil microbial diversity. ISME J. 1, 283–290. doi: 10.1038/ismej.2007.53, PMID: 18043639 PMC2970868

[ref47] Shade A. Peter H. Allison S. D. Baho D. L. Berga M. Bürgmann H.. (2012). Fundamentals of microbial community resistance and resilience. Front. Microbiol. 3:417. doi: 10.3389/fmicb.2012.00417, PMID: 23267351 PMC3525951

[ref48] Singh D. Sharma P. Kumar U. Daverey A. Daverey K. (2021). Effect of forest fire on soil microbial biomass and enzymatic activity in oak and pine forests of Uttarakhand Himalaya, India. Ecol. Process 10:29. doi: 10.1186/s13717-021-00293-6

[ref49] Stegen J. C. Lin X. Konopka A. E. Fredrickson J. K. (2012). Stochastic and deterministic assembly processes in subsurface microbial communities. ISME J. 6, 1653–1664. doi: 10.1038/ismej.2012.22, PMID: 22456445 PMC3498916

[ref50] Sundqvist M. K. Giesler R. Graae B. J. Wallander H. Fogelberg E. Wardle D. A. (2011). Interactive effects of vegetation type and elevation on aboveground and belowground properties in a subarctic tundra. Oikos 120, 128–142. doi: 10.1111/j.1600-0706.2010.18811.x

[ref51] Tian K. Kong X. Gao J. Jia Y. Lin H. He Z.. (2018). Local root status: a neglected bio-factor that regulates the home-field advantage of leaf litter decomposition. Plant Soil 431, 175–189. doi: 10.1007/s11104-018-3757-8

[ref52] Tucker C. M. Fukami T. (2014). Environmental variability counteracts priority effects to facilitate species coexistence: evidence from nectar microbes. Proc. R. Soc. B Biol. Sci. 281:20132637. doi: 10.1098/rspb.2013.2637, PMID: 24430846 PMC3906935

[ref53] Van der Putten W. H. Bardgett R. D. Bever J. D. Bezemer T. M. Casper B. B. Fukami T.. (2013). Plant-soil feedbacks: the past, the present and future challenges. J. Ecol. 101, 265–276. doi: 10.1111/1365-2745.12054

[ref54] Vannette R. L. Fukami T. (2014). Historical contingency in species interactions: towards niche-based predictions. Ecol. Lett. 17, 115–124. doi: 10.1111/ele.12204, PMID: 24341984 PMC4344821

[ref55] Wang X. Liang C. Mao J. Jiang Y. Bian Q. Liang Y.. (2023). Microbial keystone taxa drive succession of plant residue chemistry. ISME J. 17, 748–757. doi: 10.1038/s41396-023-01384-2, PMID: 36841902 PMC10119086

[ref56] Wickings K. Grandy A. S. Reed S. C. Cleveland C. C. (2012). The origin of litter chemical complexity during decomposition. Ecol. Lett. 15, 1180–1188. doi: 10.1111/j.1461-0248.2012.01837.x, PMID: 22897741

[ref57] Yuan M. M. Guo X. Wu L. Zhang Y. Xiao N. Ning D.. (2021). Climate warming enhances microbial network complexity and stability. Nat. Clim. Chang. 11, 343–348. doi: 10.1038/s41558-021-00989-9

[ref58] Zhang X. Johnston E. R. Liu W. Li L. Han X. (2016). Environmental changes affect the assembly of soil bacterial community primarily by mediating stochastic processes. Glob. Chang. Biol. 22, 198–207. doi: 10.1111/gcb.13080, PMID: 26340501

[ref59] Zhao X. Tian Q. Michelsen A. Lin Q. Yuan X. Chen L.. (2024). Home-field advantage of litter decomposition differs among leaves, absorptive roots, and transport roots. Plant Soil 1:6487. doi: 10.1007/s11104-024-06487-z

[ref60] Zhu M. Fanin N. Wang Q. Xu Z. Liang S. Ye J.. (2024). High functional breadth of microbial communities decreases home-field advantage of litter decomposition. Soil Biol. Biochem. 188:109232. doi: 10.1016/j.soilbio.2023.109232

