# Supplementary material for: Initial microbiome and tree root status structured the soil microbial community discrepancy of the subtropical pine-oak forest in a large urban forest park
Source: Front Microbiol. 2024 May 31;15:1391863. doi: 10.3389/fmicb.2024.1391863 (PMC11176443; doi:10.3389/fmicb.2024.1391863)
Supplement: Supplementary file 1 [file Presentation_1.pdf]

Supplementary Material

1 Supplementary Tables

Table S1. Sequence number and estimated alpha-diversity of the soil bacterial community for each soil sample.

Sample	Raw pairs	Trim pairs	Clean reads	Observed OTU	Chao	ACE	Shannon	Simpson	GoodsCoverage	shannoneven
PF_QS_R_1	50786	49171	42360	896	971.043	956.959	5.634	0.992	0.997	0.829
PF_QS_R_2	47027	46138	40162	933	1006.581	998.973	5.649	0.993	0.997	0.826
PF_QS_NR_1	46997	46190	40256	923	1022.167	997.294	5.650	0.992	0.996	0.828
PF_QS_NR_2	47934	47128	41401	916	1035.278	984.813	5.682	0.993	0.997	0.833
PF_PS_R_1	51171	50297	44176	890	978.065	955.504	5.629	0.992	0.997	0.829
PF_PS_NR_1	44750	43969	38102	818	915.632	885.968	5.487	0.990	0.997	0.818
PF_PS_R_2	54322	52370	44671	654	706.500	696.655	5.128	0.986	0.998	0.791
PF_PS_NR_2	43740	42959	37485	748	808.667	800.182	5.120	0.984	0.997	0.774
PF_PS_NR_3	38543	37873	32931	791	891.846	861.310	5.339	0.989	0.996	0.800

QF_QS_R_1	49950	48659	42450	912	1028.458	992.133	5.414	0.988	0.996	0.794
QF_QS_R_2	46476	45765	40478	1039	1179.457	1143.013	5.449	0.988	0.995	0.785
QF_QS_R_3	56476	55648	49326	1045	1115.011	1105.114	5.527	0.990	0.997	0.795
QF_QS_NR_1	48106	47490	42018	1000	1091.899	1063.617	5.595	0.991	0.996	0.810
QF_QS_NR_2	47482	46838	41565	984	1060.421	1049.894	5.385	0.989	0.997	0.781
QF_QS_NR_3	51317	50651	44936	1044	1141.225	1103.539	5.617	0.990	0.997	0.808
QF_PS_R_1	35407	34979	31086	947	1051.250	1030.869	5.615	0.991	0.995	0.819
QF_PS_R_2	56397	55591	49238	991	1062.842	1048.673	5.652	0.992	0.997	0.819
QF_PS_NR_1	52389	51622	45569	962	1032.012	1022.771	5.595	0.991	0.997	0.815
QF_PS_NR_2	49458	48756	43169	911	1001.718	978.992	5.502	0.990	0.997	0.807
PF_PS_R_3	41011	40261	35364	878	953.373	929.329	5.494	0.990	0.997	0.811
Average	47986.950	47117.750	41337.150	914.100	1002.672	980.280	5.508	0.990	0.997	0.809
STD	5460.198	5307.439	4715.373	100.906	110.640	107.256	0.164	0.002	0.001	0.017
CV	0.114	0.113	0.114	0.110	0.110	0.109	0.030	0.002	0.001	0.022

Table S2. Sequence number and estimated alpha-diversity of the soil fungi community for each soil sample.

Sample	Raw pairs	Trim pairs	Clean reads	Observed OTU	Chao	ACE	Shannon	Simpson	GoodsCoverage	shannoneven
PF_PS_NR_1	52343	52066	48800	96	124.500	120.140	1.714	0.624	1.000	0.376
PF_PS_NR_2	51582	50943	38550	92	92.500	92.617	2.737	0.872	1.000	0.605
PF_PS_NR_3	41794	41392	37728	41	43.500	45.158	0.328	0.102	1.000	0.088
PF_PS_R_1	37352	37061	32415	124	160.143	174.597	3.292	0.912	0.999	0.683
PF_PS_R_2	43414	43177	41592	67	77.909	80.686	0.301	0.096	1.000	0.072
PF_PS_R_3	46586	46141	40867	64	77.000	80.128	1.910	0.756	1.000	0.459
PF_QS_NR_1	47594	47289	44719	115	202.875	235.499	1.560	0.641	0.999	0.329
PF_QS_NR_2	43705	43415	40515	113	141.111	199.965	1.219	0.356	0.999	0.258
PF_QS_R_1	49450	49117	46955	114	164.000	140.764	0.868	0.368	0.999	0.183
PF_QS_R_2	50360	49982	46943	109	214.000	234.180	1.629	0.665	0.999	0.347
QF_PS_NR_1	37718	37370	32582	115	139.429	156.743	2.848	0.873	0.999	0.600

QF_PS_NR_2	36136	35868	31134	91	112.231	120.335	2.105	0.738	0.999	0.467
QF_PS_R_1	36552	36234	32906	96	123.143	143.873	2.350	0.842	0.999	0.515
QF_PS_R_2	50982	50447	43765	119	154.769	192.808	1.827	0.632	0.999	0.382
QF_QS_NR_1	49673	49281	47200	101	171.000	292.836	1.175	0.392	0.999	0.255
QF_QS_NR_2	44533	44106	42712	88	111.750	108.303	1.315	0.434	1.000	0.294
QF_QS_NR_3	44119	43571	41653	105	130.071	129.178	0.929	0.478	0.999	0.200
QF_QS_R_1	50130	49771	49040	83	117.364	153.047	0.624	0.281	0.999	0.141
QF_QS_R_2	51511	51064	51670	118	157.545	174.367	1.205	0.519	0.999	0.253
QF_QS_R_3	47479	47136	45907	122	150.111	169.270	1.825	0.640	0.999	0.380
Average	45650.650	45271.550	41882.650	98.650	133.248	152.225	1.588	0.561	0.999	0.344
STD	5409.311	5367.315	6098.361	21.893	41.793	60.128	0.813	0.245	0.000	0.172
CV	0.118	0.119	0.146	0.222	0.314	0.395	0.512	0.436	0.000	0.500

Table S3. Distance-based redundancy analysis (db-RDA) for effects of experimental effects on soil bacterial composition at phylum level.

db-RDA on Bacterial community at phylum level				
Variation		ANOVA		
Constrained	Unconstrained	Predictor	*F*	*P*
RA~original soil				
6.97%	93.03%	Soil	1.349	0.262
RA~receiving forest				
31.49%	68.51%	Forest	8.272	**0.003**
RA~root inclusion/exclusion treatment				
1.91%	98.09%	Root	0.35	0.714
RA~receiving root				
34.43%	65.57%	Root2	2.8	***0.051***
RA~original soil + receiving forest				
39.45%	60.55%	Soil	1.958	0.145
		Forest	9.119	**0.003**
RA~receiving forest + root treatment				
33.39%	66.61%	Forest	8.036	**0.006**
		Root	0.487	0.626
RA~original soil + receiving forest + root treatment				
41.36%	58.64%	Soil	1.902	0.161
		Forest	8.861	**0.004**

		Root	0.521	0.585
RA~original soil + receiving root				
42.39%	57.61%	Soil	1.816	0.171
		Root2	3.074	**0.027**

Table S4. Indicator species analysis of bacteria for each receiving root type. PN: ingrowth core incubated in PF with pine roots excluded; PR: ingrowth core incubated in PF with pine roots excluded; QN: ingrowth core incubated in QF with oak roots excluded; QR: ingrowth core incubated in QF with oak roots included.

Indicators	*A*	*B*	*IndVal*	*P*	Group
f_Gemmataceae	0.974	0.8	0.883	**0.009**	PN
g_*SM1A02*	1.000	0.6	0.775	**0.035**	PN
c_Parcubacteria	0.745	0.8	0.772	**0.043**	PN
g_*Rhodoferax*	0.767	1.0	0.876	**0.014**	QN
f_SC-I-84	0.947	0.8	0.871	**0.006**	QN
o_Saccharimonadales	0.917	0.8	0.856	**0.011**	QN
f_A21b	0.907	0.8	0.852	**0.034**	QN
f_WWH38	0.879	0.8	0.838	**0.014**	QN
o_0319-6G20	0.818	0.8	0.809	**0.019**	QN
f_Oscillospiraceae	0.806	0.8	0.803	**0.012**	QN
o_0319-6G20	1.000	0.6	0.775	**0.042**	QN
g_*Fusobacterium*	1.000	0.6	0.775	**0.036**	QN

g_*Aquicella*	1.000	0.6	0.775	**0.042**	QN
o_Group 1.1c	0.726	1.0	0.852	**0.025**	PR
g_*Edaphobaculum*	0.741	1.0	0.861	**0.039**	QR
f_Simkaniaceae	0.916	0.8	0.856	**0.014**	QR
g_*Roseiarcus*	0.694	1.0	0.833	**0.046**	QR
g_*Silvimonas*	0.656	1.0	0.81	**0.009**	QR
o_0319-6G20	0.786	0.8	0.793	**0.025**	QR
f_Lachnospiraceae	1.000	0.6	0.775	**0.047**	QR
g_*Bdellovibrio*	1.000	0.6	0.775	**0.047**	QR
g_*Coxiella*	0.867	0.6	0.721	**0.044**	QR

Table S5. Indicator species analysis of fungi for each receiving root type. PN: ingrowth core incubated in PF with pine roots excluded; PR: ingrowth core incubated in PF with pine roots excluded; QN: ingrowth core incubated in QF with oak roots excluded; QR: ingrowth core incubated in QF with oak roots included.

Indicators	*A*	*B*	*IndVal*	*P*	Group
s_*Keithomyces acicularis*	1.000	0.6	0.775	**0.035**	PN
s_*Archaeorhizomyces finlayi*	1.000	0.6	0.775	**0.026**	PN
s_*Kockovaella nitrophila*	0.995	1.0	0.997	**0.001**	QN
s_*Didymocyrtis pini*	0.973	0.8	0.882	**0.015**	QN
c_Agaricomycetes	0.776	1.0	0.881	**0.027**	QN
s_*Neovaginatispora clematidis*	1.000	0.6	0.775	**0.037**	QN

k_Fungi	1.000	0.6	0.775	**0.037**	QN
s_*Ambrosiozyma kamigamensis*	1.000	0.6	0.775	**0.034**	PR
g_*Tomentella*	0.991	1.0	0.995	**0.018**	QR
s_*Hourangia densisquamata*	0.980	0.8	0.886	**0.011**	QR
g_*Rhinocladiella*	1.000	0.6	0.775	**0.028**	QR
o_Pleosporales	1.000	0.6	0.775	**0.032**	QR

2 Supplementary Figures

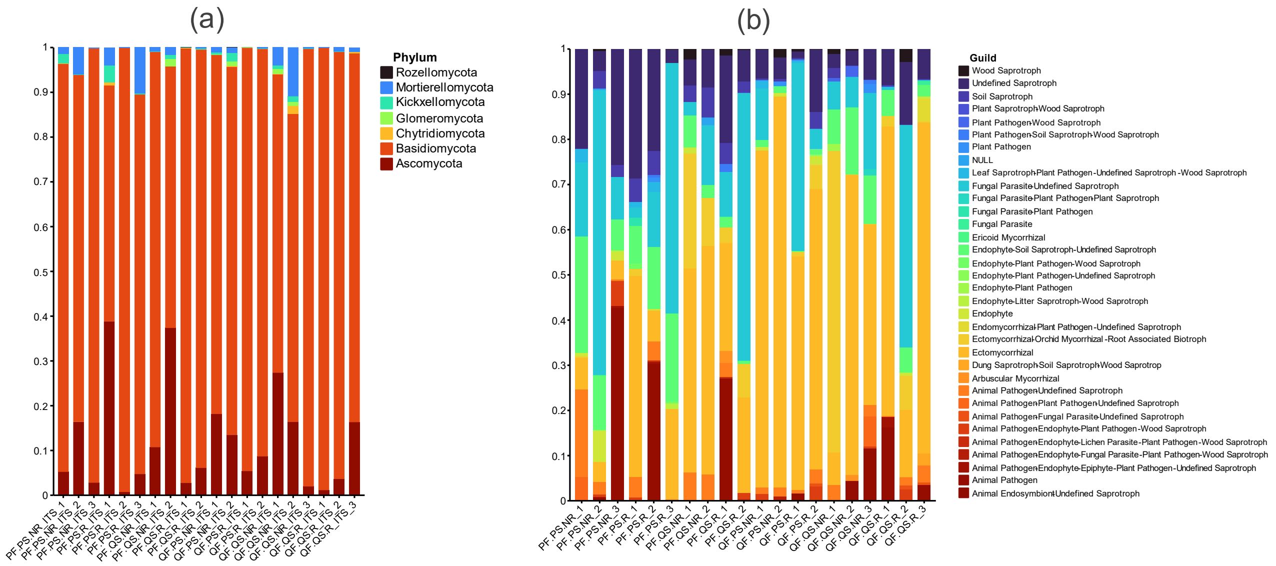

Figure S1. Relative fungi community compositions of each sample, (a) relative taxa compositions at phylum level, (b) functional compositions according to FUNGuild.
